# Mechanical power during robotic-assisted laparoscopic prostatectomy: an observational study

**DOI:** 10.1007/s10877-024-01170-1

**Published:** 2024-06-17

**Authors:** Tommaso Pozzi, Silvia Coppola, Giulia Catozzi, Andrea Colombo, Mara Chioccola, Eleonora Duscio, Fabiano Di Marco, Davide Chiumello

**Affiliations:** 1 Department of Health Sciences, University of Milan, Milan, Italy; 2 Department of Anesthesia and Intensive Care, ASST Santi Paolo e Carlo, San Paolo University Hospital Milan, Milan, Italy; 3 Coordinated Research Center On Respiratory Failure, University of Milan, Milan, Italy; 4 Pulmonary Medicine Unit, ASST Papa Giovanni XXIII, 24127 Bergamo, Italy

**Keywords:** Intraoperative mechanical ventilation, Mechanical Power, Robotic Surgery

## Abstract

**Background:**

Robotic-assisted laparoscopic radical prostatectomy (RALP) requires pneumoperitoneum and steep Trendelenburg position. Our aim was to investigate the influence of the combination of pneumoperitoneum and Trendelenburg position on mechanical power and its components during RALP.

**Methods:**

Sixty-one prospectively enrolled patients scheduled for RALP were studied in *supine position before surgery*, during *pneumoperitoneum and Trendelenburg position* and in *supine position after surgery* at constant ventilatory setting. In a subgroup of 17 patients the response to increasing positive end-expiratory pressure (PEEP) from 5 to 10 cmH_2_O was studied.

**Results:**

The application of pneumoperitoneum and Trendelenburg position increased the total mechanical power (13.8 [11.6 – 15.5] *vs* 9.2 [7.5 – 11.7] J/min, *p* < 0.001) and its elastic and resistive components compared to *supine position before surgery*. *In supine position after surgery* the total mechanical power and its elastic component decreased but remained higher compared to *supine position before surgery*. Increasing PEEP from 5 to 10 cmH_2_O within each timepoint significantly increased the total mechanical power (*supine position before surgery*: 9.8 [8.4 – 10.4] *vs* 12.1 [11.4 – 14.2] *J/min*, *p* < 0.001; *pneumoperitoneum and Trendelenburg position*: 13.8 [12.2 – 14.3] *vs* 15.5 [15.0 – 16.7] J/min, *p* < 0.001; *supine position after surgery*: 10.2 [9.4 – 10.7] *vs* 12.7 [12.0 – 13.6] J/min, *p* < 0.001), without affecting respiratory system elastance.

**Conclusion:**

Mechanical power in healthy patients undergoing RALP significantly increased both during the pneumoperitoneum and Trendelenburg position and in *supine position after surgery*. PEEP always increased mechanical power without ameliorating the respiratory system elastance.

## Introduction

Prostate cancer is the second leading cause of cancer-related death worldwide [1]. Traditionally, open radical prostatectomy has represented the only surgical option for localized prostate cancer. More recently, robotic-assisted laparoscopic radical prostatectomy (RALP) has been found to be associated with better outcomes compared to laparoscopic or open radical prostatectomy [2]. Nowadays, more than 85% of radical prostatectomies are performed by robotic-assisted procedures [3]. RALP requires a lower degree of pneumoperitoneum compared to laparoscopy, but a steep Trendelenburg position for several hours in order to facilitate surgical access [4]. Consequently, this extreme position could have a negative impact on cardio-respiratory function, especially in elderly patients and in the presence of comorbidities [5]. Regarding the respiratory function, Brandao et al. showed that pneumoperitoneum and Trendelenburg position significantly increased driving pressure due to an increase in lung and – to a greater extension—chest wall elastance [6]. In addition the functional residual capacity and the respiratory system compliance and its components (*i.e.* lung and chest wall) might decrease up to 50% compared to pneumoperitoneum in supine position promoting lung atelectasis and derecruitment [7, 8].

Similarly to open surgery, it has been suggested to apply a lung protective ventilatory strategy, with low tidal volume, low driving pressure and higher positive end-expiratory pressure (PEEP), also during RALP, to minimize lung stress and reduce postoperative complications [5, 9–13]. However, previous randomized clinical studies evaluating the effects of intraoperative lung protective ventilation in a non-robotic setting have showed conflicting results [5, 14]. Therefore, it has been proposed that the extent of ventilator-induced lung injury (VILI) may be more related to the total amount of energy delivered from the ventilator to the patient, defined as mechanical power [15, 16]. Mechanical power depends on tidal volume, respiratory rate, driving pressure, inspiratory flow and PEEP [16, 17] as well as on the intrinsic respiratory system characteristics of the patient. Two *post-hoc* analyses of large randomized trials and a retrospective study of patients undergoing open abdominal surgery found that a higher mechanical power was significantly associated with a greater risk of postoperative respiratory failure [18–20]. However, the influence of the steep Trendelenburg position with pneumoperitoneum on mechanical power and its components (*i.e.* the elastic and resistive) in patients undergoing RALP has not been evaluated. The aim of this study was to investigate the influence of the combination of the steep Trendelenburg position and pneumoperitoneum during RALP on respiratory mechanics, particularly mechanical power, gas exchange and hemodynamics.

## Materials and Methods

### Study design

Prospective non-randomized study. All consecutive adult patients scheduled for RALP with the da Vinci surgical system at San Paolo University Hospital, Milan, Italy, were considered eligible for the study. Exclusion criteria were: American Society of Anesthesiologists (ASA) score equal to or higher than 3, history of severe chronic obstructive pulmonary disease, previous lung surgery. The study was approved by the Institutional Review board of our hospital (Comitato Etico Interaziendale Milano Area A, 2023/ST/057) and written informed consent was obtained according to the Italian regulations before the beginning of the surgery.

### Anesthesia and surgical protocol

General anaesthesia and paralysis were induced with Propofol 2 mg/kg, Fentanyl 2 mcg/kg and Rocuronium 1 mg/kg. Balanced general anaesthesia was performed with a continuous infusion of Rocuronium of 0.2 mg/kg/h to ensure a bispectral index score (BIS) value between 45–55 and a post tetanic count of less than 8, respectively. Patients were ventilated using volume-controlled ventilation with a tidal volume between 8–10 ml/kg ideal body weight, an inspiratory ratio of 1:2 and a PEEP of 5 cmH_2_O. Respiratory rate was adjusted to achieve an end-tidal carbon dioxide partial pressure (EtCO_2_) between 35–45 mmHg. A fluid infusion of 2 ml/kg/h of crystalloid was maintained throughout the procedure. Pneumoperitoneum was induced using an Air Seal insufflation system (ConMed, Drogenbos, Belgium) with an intraperitoneal pressure of 10 mmHg. Lithotomy with a steep Trendelenburg position (head down between 26–30 degree) was performed when the surgeon declared ready to start the operative phase.

### Data collection

Demographic and clinical data were collected from the hospital medical records. Intraoperative data were recorded 10 min after the intubation, in the supine position (*supine position before surgery* timepoint), 10 min after induction of pneumoperitoneum and Trendelenburg position after the docking (*pneumoperitoneum and Trendelenburg position* timepoint) and 10 min after return to supine position with pneumoperitoneum at the end of the surgery (*supine position after surgery* timepoint). In a subgroup of patients, after each measurement timepoint at 5 cmH_2_O of PEEP, positive end-expiratory pressure was transiently increase at 10 cmH_2_O for 10 min to evaluate the effects of two PEEP levels on respiratory mechanics, gas exchange and hemodynamics. A low (5 cmH_2_O) and high (10 cmH_2_O) level of PEEP were applied as extremes levels to assess the possible beneficial or detrimental effects. The reported values are the average of 3 measurements obtained within 2 min at each measurement timepoint.

### Measurements

At each measurement timepoint, the following variables were recorded:

- Respiratory mechanics: airway peak inspiratory pressure, airway plateau pressure after a 5-s end-inspiratory hold and total PEEP after a 5-s end-expiratory hold were recorded.
- Hemodynamics: Systolic, diastolic and mean arterial pressure and heart rate.
- Peripheral oxygen saturation and EtCO_2_.

### Calculations of mechanical power

Mechanical power (MP) was computed according to a previously described simplified formula:

$$MP=0.098\times Vt\times RR\times (Peak \;Pressure-\frac{Driving \;Pressure}{2})$$

where Vt is tidal volume, RR is respiratory rate and driving pressure is computed as the difference between airway plateau pressure and total PEEP. Mechanical power was also partitioned in the resistive (MP_RES_) and elastic (MP_EL_) components, as follows:

$${MP}_{RES}=0.098\times Vt\times RR\times (Peak \;Pressure-Plateau \;Pressure)$$

$${MP}_{EL}=0.098\times Vt\times RR\times (\frac{Driving \;Pressure}{2})$$

Mechanical power ratio was calculated as the ratio between measured and ideal mechanical power; the latter was derived from a previously published linear regression in a population of heathy patients undergoing general anesthesia, accounting for age, sex and height [21].

### Statistical analysis

From a preliminary analysis, we calculated that a sample size of 50 patients would have provided the study a power of 0.80 with a confidence level of 0.05 to detect a variation of mechanical power of 30% within measurement timepoints. Continuous variables are reported as median [IQR], while categorical variables as % (number). Differences within measurement timepoints were assessed by One-Way Repeated Measures ANOVA or Friedmann Test, as appropriate. In a subgroup of patients, differences within measurements timepoint and PEEP were assessed by Two-Ways Repeated Measures ANOVA or mixed linear effect model, assuming timepoint and PEEP as fixed *within* effect and the patient as a random effect. Multiple comparisons were performed with pairwise Student’s T test or Wilcoxon-Mann–Whitney U test with Bonferroni correction, as appropriate. A *p* value < 0.05 was considered statistically significant.

## Results

Eighty patients were prospectively screened for eligibility; after excluding 19 patients, 61 patients were enrolled; in a subgroup of 17 patients two levels of PEEP were tested at each timepoint. Their main clinical characteristics are shown in Table 1. The median BMI was 26 [24 – 26] kg/m^2^ and 5 (8%) of the patients had a BMI higher than 30 kg/m^2^. The applied tidal volume was 9.9 [9.3 – 10.5] mL/kg of predicted body weight with 5 cmH_2_O of PEEP, resulting in a driving pressure of 8 [6﻿ – 10] cmH_2_O and a respiratory system elastance of 15 [12﻿ – 18] cmH_2_O/L. The total mechanical power was 9.2 [7.5 – 11.7] J/min, with a resistive and elastic component of 3.0 [2.1 – 4.7] and 6.1 [5.1 – 7.4] J/min, respectively.

**Table 1:** Baseline characteristics of the study population. BMI: body mass index; ASA: American Society of Anesthesiologists

	n = 61
Age, *years*	68 [64 – 74]
Weight, *kg*	78 [73 – 83]
Height, *cm*	175 [168 – 180]
BMI, kg/m^2^	26 [24 – 26]
BMI classification, *% (n)*	
< 18.5	2 (1)
18.5 – 25.0	38 (23)
25.0 – 30.0	52 (32)
> 30.0	8 (5)
ASA classification, *% (n)*	
1	10 (6)
2	77 (47)
3	13 (8)
Comorbidities, *% (n)*	
Hypertension	46 (28)
Diabetes	11 (7)
Chronic artery disease	8 (5)
Cerebrovascular disease	5 (3)
Total anesthesia time, *min*	285 [255 – 337]

### Effects of pneumoperitoneum and Trendelenburg position

**Fig. 1:**
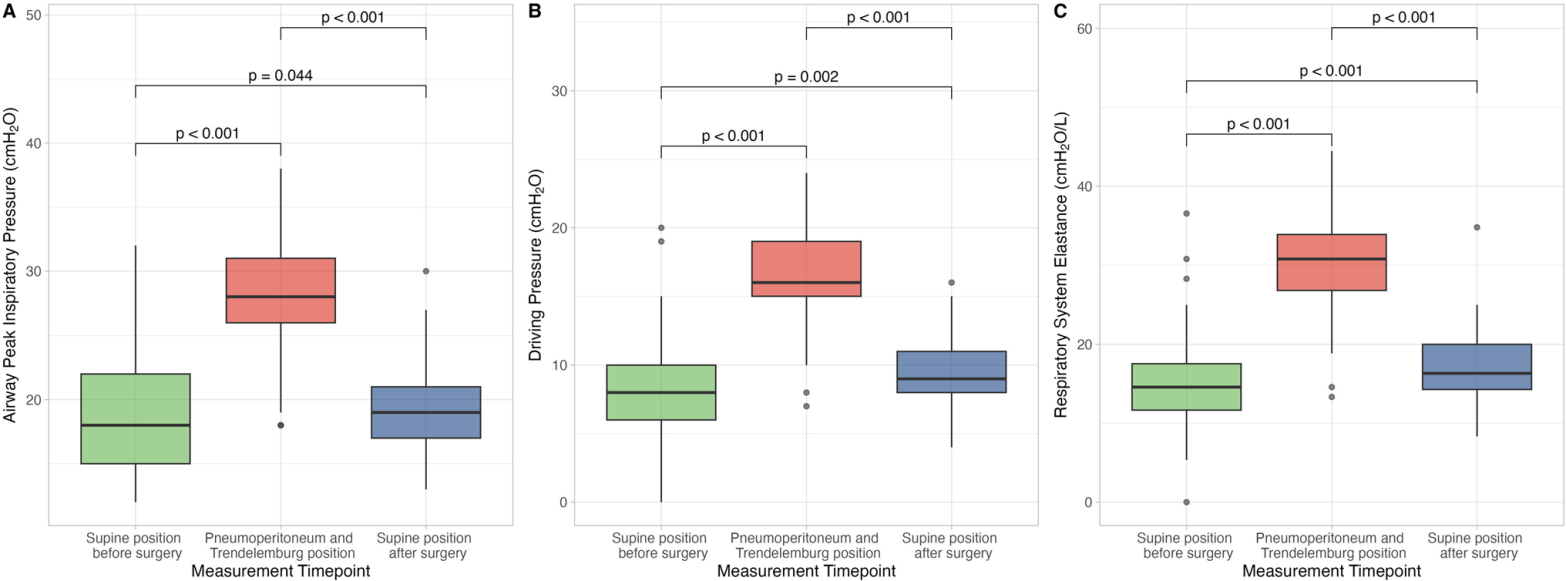
Peak inspiratory pressure (**A**) and plateau pressure (**B**) among measurement timepoints

The applied tidal volume did not change among the three timepoints, whereas the respiratory rate was slightly higher during pneumoperitoneum and Trendelenburg and after surgery compared to *supine position before surgery* timepoint (Table 2). Airway peak inspiratory pressure, driving pressure and respiratory system elastance were significantly higher during pneumoperitoneum and Trendelenburg compared to *supine position before surgery* timepoint (Fig. 1). Total mechanical power, as well as the resistive and elastic components, were significantly higher during pneumoperitoneum and Trendelenburg compared to *supine position before surgery* timepoint (Table 2).

**Table 2:** Respiratory mechanics, gas exchange and hemodynamics according to measurement timepoint. PBW: predicted body weight; PEEP: positive end-expiratory pressure; EtCO_2_: end-tidal carbon dioxide partial pressure; SpO_2_: peripheral oxygen saturation. One-way repeated measures ANOVA or Friedman Test with *post-hoc* multiple comparisons. *: *p* < 0.05 *vs supine before surgery* timepoint; °: *p* < 0.05 *vs Pneumoperitoneum and Trendelenburg position*. Statistical results for tidal volume and tidal volume per PBW are omitted, as tidal volume is kept constant throughout the whole study

	*Supine position before surgery*	*Pneumoperitoneum* *and Trendelenburg position*	*Supine position after surgery*	*p*
Tidal volume, *mL*	550 [510 – 600]	550 [510 – 600]	550 [510 – 600]	*-*
Tidal volume per PBW, *mL/kg*	9.9 [9.3 – 10.5]	9.9 [9.2 – 10.7]	9.8 [9.1 – 10.7]	*-*
Respiratory rate, *bpm*	12 [12-14]	13 [12-14]*	13 [12-14]*	*0.001*
Minute ventilation, *L/min*	6.8 [6.2 – 7.6]	7.1 [6.6 – 7.8]*	7.2 [6.7 – 7.8]*	*0.001*
Peak inspiratory pressure, *cmH*_*2*_*O*	18 [15-22]	28 [26-31]	19 [17-21]*°	*0.001*
Plateau pressure, *cmH*_*2*_*O*	13 [11-15]	23 [4, 11, 20-23]^*^	15 [13-16]*°	*0.001*
Driving pressure, *cmH*_*2*_*O*	8 [6-10]	16 [15-19]*	9 [8-11]*°	*0.001*
Respiratory system elastance, *cmH*_*2*_*O/L*	15 [12-18]	31 [25-32]*	16 [14-20]*°	*0.001*
Airway resistance, *cmH*_*2*_*O/L/sec*	13 [11-17]	15 [11-22]*	13 [8-19]°	*0.001*
Mechanical power, *J/min*	9.2 [7.5 – 11.7]	13.8 [11.6 – 15.5]*	10.0 [8.5 – 11.9]*°	*0.001*
Resistive mechanical power, *J/min*	3.0 [2.1 – 4.7]	4.0 [2.6 – 5.6]*	3.3 [1.8 – 4.6]°	*0.001*
Elastic mechanical power, *J/min*	6.1 [5.1 – 7.4]	9.6 [8.3 – 11.0]*	7.0 [6.0 – 7.9]*°	*0.001*
Mechanical power ratio	1.0 [0.9 – 1.4]	1.6 [1.4 – 1.9]*	1.2 [1.0 – 1.4]*°	*0.001*
EtCO_2_, *mmHg*	34 [30-35]	36 [31-37]*	35 [31-33]*°	*0.001*
SpO_2_, *%*	99 [98 – 100]	98 [96 – 99]*	98 [97 – 100]	*0.001*
Systolic arterial pressure, *mmHg*	113 [102 – 130]	125 [119 – 136]*	113 [100 – 130]°	*0.032*
Diastolic arterial pressure, *mmHg*	69 [62 – 74]	78 [69 – 86]*	65 [56 – 73]°	*0.001*
Mean arterial pressure, *mmHg*	82 [74 – 91]	95 [86 – 103]*	81 [70 – 90]°	*0.001*
Heart rate, *bpm*	63 [56 – 71]	60 [56 – 69]	61 [58 – 66]	*0.500*

In *supine position after surgery* timepoint, airway peak inspiratory pressure, driving pressure and mechanical power were significantly lower compared to pneumoperitoneum and Trendelenburg. However, airway peak inspiratory pressure, driving pressure and respiratory system elastance were significantly higher compared to *supine position before surgery timepoint*. Partitioning mechanical power into elastic and resistive components, both significantly increased during pneumoperitoneum and Trendelenburg position compared to *supine position before surgery*; both the resistive and the elastic components decreased in *supine position after surgery* timepoint, but only the resistive component returned to basal value. Considering mechanical power ratio, it was significantly higher during pneumoperitoneum and Trendelenburg compared to *supine position before* and *after surgery* timepoints (1.6 [1.4 – 1.9] *vs* 1.0 [0.9 – 1.4] or 1.2 [1.0 – 1.4], *p* = 0.001). Although minute ventilation was significantly higher in pneumoperitoneum and Trendelenburg timepoint, EtCO_2_ was significantly higher compared to *supine position before surgery*. However, EtCO_2_ remained significantly higher in *supine position after surgery* timepoint.

### Effects of increasing PEEP within timepoints

Both at 5 and at 10 cmH_2_O of PEEP, comparing pneumoperitoneum and Trendelenburg position to *supine position* both *before* and *after surgery* timepoints*,* airway peak inspiratory pressure, driving pressure, respiratory system elastance and mechanical power were significantly higher in pneumoperitoneum and Trendelenburg position (Table 3).

**Table 3:** Respiratory mechanics, gas exchange and hemodynamics according to measurement timepoints and PEEP levels. PBW: predicted body weight; PEEP: positive end-expiratory pressure; EtCO_2_: end-tidal carbon dioxide partial pressure; SpO_2_: peripheral oxygen saturation. Two-ways repeated measures ANOVA with *post-hoc* multiple comparisons. *: *p* < 0.05 *vs supine position before surgery* timepoint; °: *p* < 0.05 *vs Pneumoperitoneum and Trendelemburg position*; ^§^: *p* < 0.05 *vs* PEEP 5 cmH_2_O

	*Supine position before surgery*	*Pneumoperitoneum and Trendelenburg position*	*Supine position after surgery*	*p*_*TIME*_	*p*_*PEEP*_	*p*_*INT*_
Peak pressure, *cmH*_*2*_*O*						
5 cmH_2_O of PEEP	19 [17-22]	28 [25-28]*	20 [18-22]°	***0.001***	***0.001***	*0.484*
10 cmH_2_O of PEEP	23 [ 21-25]^§^	31 [27-31]*^§^	24 [11, 22, 23]°^§^			
Plateau pressure, *cmH*_*2*_*O*						
5 cmH_2_O of PEEP	14 [12-16]	22 [4, 11, 20-23]*	16 [14-17]°	***0.001***	***0.001***	*0.318*
10 cmH_2_O of PEEP	19 [16-20]^§^	25 [4, 11, 24-26]*^§^	19 [17-20]°^§^			
Driving pressure, *cmH*_*2*_*O*						
5 cmH_2_O of PEEP	8 [7-11]	16 [15-20]*	11 [9-11]*°	***0.001***	*0.079*	*0.278*
10 cmH_2_O of PEEP	9 [6-10]	15 [14-18]*	9 [7-10]°			
Respiratory system elastance, *cmH*_*2*_*O/L*						
5 cmH_2_O of PEEP	15 [12-21]	33 [26-34]*	19 [16-23]*°	***0.001***	*0.070*	*0.286*
10 cmH_2_O of PEEP	15 [12-20]	29 [4, 24-32]*	16 [13-18]°			
Airway resistance, *cmH*_*2*_*O/L/sec*						
5 cmH_2_O of PEEP	13 [12-17]	16 [10-20]	14 [8-19]	*0.571*	*0.398*	*0.624*
10 cmH_2_O of PEEP	12 [11-16]	14 [11-18]	13 [8-18]			
Mechanical power, *J/min*						
5 cmH_2_O of PEEP	9.8 [8.4 – 10.4]	13.8 [12.2 – 14.3]*	10.2 [9.4 – 10.7]°	***0.001***	***0.001***	*0.871*
10 cmH_2_O of PEEP	12.1 [11.4 – 14.2]^§^	15.5 [15.0 – 16.7]*^§^	12.7 [12.0 – 13.6]°^§^			
Resistive mechanical power, *J/min*						
5 cmH_2_O of PEEP	2.7 [2.4 – 3.7]	3.7 [2.3 – 5.3]	3.3 [1.6 – 4.0]	*0.320*	*0.528*	*0.655*
10 cmH_2_O of PEEP	2.7 [2.4 – 3.1]	3.5 [2.3 – 4.3]	2.6 [2.2 – 4.0]			
Elastic mechanical power, *J/min*						
5 cmH_2_O of PEEP	6.5 [5.8 – 7.1]	9.1 [8.3 – 11.2]*	7.0 [6.3 – 7.9]°	***0.001***	***0.001***	*0.528*
10 cmH_2_O of PEEP	9.7 [9.0 – 10.8]^§^	12.7 [11.2 – 13.5]*^§^	9.7 [8.9 – 10.5]°^§^			
Mechanical power ratio						
5 cmH_2_O of PEEP	1.1 [0.9 – 1.3]	1.6 [1.4 – 1.7]*	1.2 [1.0 – 1.3]°	***0.001***	***0.001***	*0.883*
10 cmH_2_O of PEEP	1.4 [1.3 – 1.7]^§^	1.8 [1.7 – 2.0]*^§^	1.5 [1.4 – 1.6]°^§^			
EtCO_2_, *mmHg*						
5 cmH_2_O of PEEP	34 [30-35]	36 [33-35]	36 [33-38]*	***0.011***	*0.514*	*0.808*
10 cmH_2_O of PEEP	34 [30-35]	36 [32-36]	36 [33-37]*			
SpO_2_, *%*						
5 cmH_2_O of PEEP	99 [98 – 100]	99 [98 – 100]	99 [98 – 100]	*0.113*	*0.866*	*0.891*
10 cmH_2_O of PEEP	99 [98 – 100]	99 [98 – 99]	99 [98 – 100]			
Systolic arterial pressure, *mmHg*						
5 cmH_2_O of PEEP	112 [101 – 127]	126 [117 – 132]*	110 [101 – 130]	***0.021***	*0.269*	*0.946*
10 cmH_2_O of PEEP	110 [98 – 120]	120 [107 – 130]*	111 [101 – 127]			
Diastolic arterial pressure, *mmHg*						
5 cmH_2_O of PEEP	72 [69 – 76]	76 [69 – 86]	66 [55 – 73]°	***0.001***	*0.179*	*0.727*
10 cmH_2_O of PEEP	68 [59 – 74]	73 [68 – 88]	66 [53 – 70]°			
Mean arterial pressure, *mmHg*						
5 cmH_2_O of PEEP	88 [79 – 90]	92 [85 – 101]	82 [69 – 92]°	***0.001***	*0.485*	*0.948*
10 cmH_2_O of PEEP	82 [73 – 90]	90 [80 – 102]	78 [70 – 89]°			
Heart rate, *bpm*						
5 cmH_2_O of PEEP	63 [60 – 80]	60 [58 – 69]	62 [59 – 68]	*0.497*	*0.454*	*0.310*
10 cmH_2_O of PEEP	61 [60 – 80]	60 [54 – 72]	62 [57 – 66]			

Comparing the effects of increasing PEEP within each of the timepoints, 10 cmH_2_O of PEEP significantly increased airway peak inspiratory pressure, airway plateau pressure and total and elastic mechanical power compared to 5 cmH_2_O of PEEP, while driving pressure and respiratory system elastance did not change compared to 5 cmH_2_O of PEEP (Table 3). Increasing PEEP within each time point increased the elastic component of mechanical power, while did not affect the resistive component.

## Discussion

The main findings of this study are: 1) pneumoperitoneum and Trendelenburg position increased respiratory system elastance, total mechanical power and mechanical power ratio compared to *supine position before* and *after surgery*; 2) respiratory system elastance and total mechanical power were significantly higher *in supine position after surgery* compared to *supine position before surgery* timepoint and 3) the application of 10 cmH_2_O of PEEP compared to 5 cmH_2_O in *pneumoperitoneum and Trendelenburg position* significantly increased mechanical power, although not affecting respiratory system elastance.

Nowadays, RALP has become the most common procedure for prostate cancer due to a more precise dissection and to better recovery after surgery [1, 2]. However, in addition to pneumoperitoneum, RALP requires the use of a steep Trendelenburg position, which could impair the respiratory, cardiovascular and central nervous system [5]. In particular, the use of pneumoperitoneum and of a steep Trendelenburg position promote a decrease in lung and chest wall compliance at different extents, by increasing intrathoracic pressure and moving the diaphragm to a more cranial position [22, 23]. These negative effects could decrease lung gas volume (*i.e.* functional residual capacity), promote hypoxaemia, increase stress and strain, driving pressure, thus promoting ventilator-induced lung injury, and increase postoperative pulmonary complication rates. Therefore, it has been recommended to avoid intrabdominal pressure higher than 12 mmHg during pneumoperitoneum and Trendelenburg position to minimize the negative respiratory effects [4, 11].

Previous studies reported that a higher intraoperative driving pressure during general anesthesia could promote postoperative pulmonary complications [10, 24]. However, during controlled ventilation, tidal volume, inspiratory flow, respiratory rate and PEEP also contribute to VILI, in addition to driving pressure [16]. In passive conditions, according to the equation of motion, the pressure required to inflate the patient is used to overcome the resistive and elastic components of the respiratory system. When the product of this pressure for the inflated volume is calculated for every breath in one minute, it is defined as mechanical power. Mechanical power is thus a unifying tool that takes into account all the components of the ventilation (tidal volume, respiratory rate, driving pressure, inspiratory flow and PEEP), along with the intrinsic characteristics of the lung [16, 17]. Previous experimental data in animal and in ARDS patients showed that VILI and outcome were related to the amount of mechanical power [15, 25, 26]. More recently in surgical patients under general anesthesia, mechanical power was also found to be associated to postoperative pulmonary complications and reintubation [18–20]. However, it is not clear if a intraoperative ventilatory strategy based on mechanical power could improve outcomes. Additionally, simpler indices have been proposed [27].

In the present study, we focused only on RALP and on the changes in total mechanical power and its components. Total mechanical power increased significantly during *pneumoperitoneum and Trendelenburg position* as compared to the *supine position*, both *before* and *after surgery*, mainly due to the changes in the intrinsic properties of the respiratory system. The observed increase in mechanical power was similar to a previous study in patients undergoing major non-cardiothoracic non-neurosurgical surgery, in which the computed median value was 9 [7–0 to 11.4] J/min [18–20]—obviously lower than in ARDS patients [25]. At present, there is no clear threshold for mechanical power as a risk factor in modulating VILI during general surgery compared to ARDS patients [25]. In healthy patients during general anesthesia every increase of 5 J/min in mechanical power increased the odds of reintubation by 31% [19]. In the present study, we tried to better evaluate the changes in mechanical power in a standardized way through mechanical power ratio, computed as the ratio between the actual mechanical power and the expected mechanical power given similar patient’s baseline characteristics. Mechanical power ratio increased with pneumoperitoneum and Trendelenburg position, but always remained below 2.0. This small increase in mechanical power ratio in healthy patients during a relatively short period of general anaesthesia was not associated with major pulmonary complications, such as reintubation, which never occurred in our study population.

In addition, mechanical power is constituted of two components: 1) the power to overcome the airway resistance (*i.e.* resistive mechanical power), and 2) the power to inflate the respiratory system during each breath and to maintain airway inflation for a given PEEP (*i.e.* elastic mechanical power). Resistive mechanical power is the amount of energy dissipated during the inspiration and is mainly determined by the patient’s resistance and inspiratory flow. The observed increase in resistive mechanical power during pneumoperitoneum and Trendelenburg position was mainly due to a slight increase in respiratory rate, which increased inspiratory flow. Similarly, during robotic-assisted abdominal surgery, resistive mechanical power was shown to increase during pneumoperitoneum as compared to supine position; this increase was significantly higher in obese compared to lean subjects [28]. The potential role of resistive mechanical power in modulating the VILI is currently unknown [29]. The elastic component of mechanical power increased significantly during *pneumoperitoneum and Trendelenburg position* while keeping tidal volume constant throughout the study, due to an increase in respiratory system elastance by approximately twice the baseline value.

Although lung protective ventilatory strategy suggests the application of a PEEP level, optimal PEEP and the technique to individualize it in each patient remains controversial. During mechanical ventilation, PEEP levels usually do not exceed 5 cmH_2_O, which can be not sufficient to avoid lung atelectasis and prevent VILI [22]. Previous studies suggested to select PEEP achieving the maximal respiratory system compliance, the lower driving pressure or according to the intrabdominal pressure [9, 22, 30–32]. When PEEP was individualized, resulting in levels between 12–14 cmH_2_O, arterial oxygenation and respiratory system compliance were higher compared to a lower fixed level (*i.e.* 5 cmH_2_O) [22, 31, 33, 34]. However, in the same position, increasing PEEP to 10 cmH_2_O raised total mechanical power compared to 5 cmH_2_O of PEEP, although respiratory system elastance did not change. Thus, at the same time, the increase in PEEP caused a decrease in driving pressure while increasing the total and the elastic component of mechanical power. Without partitioning mechanical power at different levels of PEEP, it could not have been possible to evaluate the differential effect of PEEP on elastic and resistive components. Thus, although the increase of PEEP may have beneficial effects in the short term - mainly on oxygenation -, it should be balanced by an increase in mechanical power, with a higher requirement of fluids and hemodynamic impairment [15]. Of note, previous studies demonstrated that PEEP levels of 10 cmH_2_O or lower were not associated with relevant hemodynamic effects [35].

After the patient returned in supine position, a significant increase in respiratory elastance was found, probably due to basal lung atelectasis occurring in Trendelenburg position [7, 36]. The use of pneumoperitoneum, especially when applied for several hours, can lead to CO_2_ absorption, promoting hypercapnia and acidemia. To prevent acidemia, the titration of minute ventilation by close intraoperative monitoring of EtCO_2_ is necessary. In order to improve the surgery field and reduce CO_2_ absorption as much as possible, the AirSeal system was used. This system is able to reduce the CO_2_ flow up to 3 L/min during pneumoperitoneum when the set pressure is reached [37, 38]. During RALP, arterial oxygenation and carbon dioxide can be affected due to ventilation-perfusion mismatch, increased shunt and dead space [7]. Typically, the increase in arterial CO_2_ is due to increased CO_2_ production (the effect of CO_2_ insufflation for the pneumoperitoneum) and decreased CO_2_ elimination from the lung. Usually, clinicians increase minute ventilation to avoid a significant increase in CO_2_. In the present study, EtCO_2_ did not return to its baseline value at the end of the pneumoperitoneum; this could be due to a reduction of CO_2_ elimination, as a result of an increase in ventilation-perfusion mismatch and a significant amount of CO_2_ stored in the extravascular compartment during surgery, which is subsequently released [37].

## Limitations

The induction of pneumoperitoneum with or without Trendelenburg position are known factors that promote an increase in chest wall and lung elastance at different extents. Unfortunately, in the present study we did not assess the transpulmonary pressure, so it was not possible to understand whether the increase in respiratory system elastance was mainly due to chest wall or lung stiffness increase.

## Conclusions

Calculation of mechanical power, which can be obtained by assessing common indicators of mechanical ventilation, provides more information than individual ventilatory parameters. Mechanical power in healthy patients undergoing RALP significantly increased both with pneumoperitoneum in Trendelenburg position and in supine position after surgery. Positive end-expiratory pressure always increased mechanical power without ameliorating the respiratory system elastance.

## Glossary of terms

RALP: Robotic-assisted laparoscopic radical prostatectomy
PEEP: Positive end-expiratory pressure
BIS: Bispectral index score
VILI: Ventilator-induced lung injury
EtCO_2_: End-tidal carbon dioxide partial pressure
Vt: Tidal volume
RR: Respiratory rate
MP: Mechanical power
MP_RES_: Resistive mechanical power component
MP_EL_: Elastic mechanical power component

## References

[1] Basiri A, de la Rosette JJ, Tabatabaei S, Woo HH, Laguna MP, Shemshaki H. Comparison of retropubic, laparoscopic and robotic radical prostatectomy: who is the winner? World J Urol. 2018;36:609–21. 29362896 10.1007/s00345-018-2174-1

[2] Du Y, Long Q, Guan B, Mu L, Tian J, Jiang Y, et al. Robot-Assisted Radical Prostatectomy Is More Beneficial for Prostate Cancer Patients: A System Review and Meta-Analysis. Med Sci Monit. 2018;24:272–87. 29332100 10.12659/MSM.907092 PMC5776881

[3] Carbonara U, Srinath M, Crocerossa F, Ferro M, Cantiello F, Lucarelli G, et al. Robot-assisted radical prostatectomy versus standard laparoscopic radical prostatectomy: an evidence-based analysis of comparative outcomes. World J Urol. 2021;39:3721–32. 33843016 10.1007/s00345-021-03687-5

[4] Autorino R, Porpiglia F. Robotic surgery in urology: the way forward. World J Urol. 2020;38:809–11. 32189091 10.1007/s00345-020-03163-6

[5] Aceto P, Galletta C, Cambise C, et al. Challenges for anaesthesia for robotic-assisted surgery in the elderly. Europ J Anaesthesiol Intensive Care. 2023;2:e0019. 10.1097/EA9.0000000000000019 PMC11783671 39917591

[6] Brandão JC, Lessa MA, Motta-Ribeiro G, et al. Global and Regional Respiratory Mechanics During Robotic-Assisted Laparoscopic Surgery: A Randomized Study. Anesth Analg. 2019;129:1564–73.31743177 10.1213/ANE.0000000000004289

[7] Kalmar AF, Foubert L, Hendrickx JFA, et al. Influence of steep Trendelenburg position and CO2 pneumoperitoneum on cardiovascular, cerebrovascular, and respiratory homeostasis during robotic prostatectomy. Br J Anaesth. 2010;104:433–9. 20167583 10.1093/bja/aeq018

[8] Lestar M, Gunnarsson L, Lagerstrand L, et al. Hemodynamic Perturbations During Robot-Assisted Laparoscopic Radical Prostatectomy in 45° Trendelenburg Position. Anesth Analg. 2011;113:1069–75. 21233502 10.1213/ANE.0b013e3182075d1f

[9] Young CC, Harris EM, Vacchiano C, et al. Lung-protective ventilation for the surgical patient: international expert panel-based consensus recommendations. Br J Anaesth. 2019;123:898–913. 31587835 10.1016/j.bja.2019.08.017

[10] Ladha K, Vidal Melo MF, McLean DJ, Wanderer JP, Grabitz SD, Kurth T, Eikermann M. Intraoperative protective mechanical ventilation and risk of postoperative respiratory complications: hospital based registry study. BMJ. 2015;351:h3646. 10.1136/bmj.h3646. 10.1136/bmj.h3646 PMC4501577 26174419

[11] Corcione F, Esposito C, Cuccurullo D, Settembre A, Miranda N, Amato F, et al. Advantages and limits of robot-assisted laparoscopic surgery: preliminary experience. Surg Endosc. 2005;19:117–9. 15549629 10.1007/s00464-004-9004-9

[12] Güldner A, Kiss T, Serpa Neto A, et al. Intraoperative Protective Mechanical Ventilation for Prevention of Postoperative Pulmonary Complications. Anesthesiology. 2015;123:692–713. 26120769 10.1097/ALN.0000000000000754

[13] Suleiman A, Costa E, Santer P, et al. Association between intraoperative tidal volume and postoperative respiratory complications is dependent on respiratory elastance: a retrospective, multicentre cohort study. Br J Anaesth. 2022;129:263–72. 35690489 10.1016/j.bja.2022.05.005 PMC9837741

[14] Hol L, Nijbroek SGLH, Schultz MJ. Perioperative Lung Protection: Clinical Implications. Anesth Analg. 2020;131:1721–9. 33186160 10.1213/ANE.0000000000005187

[15] Collino F, Rapetti F, Vasques F, et al. Positive End-expiratory Pressure and Mechanical Power. Anesthesiology. 2019;130:119–30. 30277932 10.1097/ALN.0000000000002458

[16] Gattinoni L, Tonetti T, Cressoni M, Cadringher P, Herrmann P, Moerer O, et al. Ventilator-related causes of lung injury: the mechanical power. Intensive Care Med. 2016;42:1567–75. 27620287 10.1007/s00134-016-4505-2

[17] Chiumello D, Gotti M, Guanziroli M, Formenti P, Umbrello M, Pasticci I, et al. Bedside calculation of mechanical power during volume- and pressure-controlled mechanical ventilation. Crit Care. 2020;24:417. 32653011 10.1186/s13054-020-03116-w PMC7351639

[18] Schuijt MTU, Hol L, Nijbroek SG, et al. Associations of dynamic driving pressure and mechanical power with postoperative pulmonary complications–posthoc analysis of two randomised clinical trials in open abdominal surgery. EClinicalMedicine. 2022;47: 101397. 35480074 10.1016/j.eclinm.2022.101397 PMC9035701

[19] Santer P, Wachtendorf LJ, Suleiman A, et al. Mechanical Power during General Anesthesia and Postoperative Respiratory Failure: A Multicenter Retrospective Cohort Study. Anesthesiology. 2022;137:41–54. 35475882 10.1097/ALN.0000000000004256

[20] Karalapillai D, Weinberg L, Neto AS, et al. Intra-operative ventilator mechanical power as a predictor of postoperative pulmonary complications in surgical patients. Eur J Anaesthesiol. 2022;39:67–74. 34560687 10.1097/EJA.0000000000001601 PMC8654268

[21] Fratti I, Pozzi T, Corrente M, Galetta G, Lopez P, Grieco S, et al. Effect of PEEP variation on mechanical power in healthy patients during general anesthesia. ESCIM Abstract Collection. 2023.

[22] Girrbach F, Petroff D, Schulz S, et al. Individualised positive end-expiratory pressure guided by electrical impedance tomography for robot-assisted laparoscopic radical prostatectomy: a prospective, randomised controlled clinical trial. Br J Anaesth. 2020;125:373–82. 32665059 10.1016/j.bja.2020.05.041

[23] Stolzenburg J-U, Kallidonis P, Qazi H, Ho Thi P, Dietel A, Liatsikos EN, et al. Extraperitoneal Approach for Robotic-assisted Simple Prostatectomy. Urology. 2014;84:1099–105. 25443912 10.1016/j.urology.2014.06.045

[24] Neto AS, Hemmes SNT, Barbas CSV, Beiderlinden M, Fernandez-Bustamante A, Futier E, et al. Association between driving pressure and development of postoperative pulmonary complications in patients undergoing mechanical ventilation for general anaesthesia: a meta-analysis of individual patient data. Lancet Respir Med. 2016;4:272–80. 26947624 10.1016/S2213-2600(16)00057-6

[25] Coppola S, Caccioppola A, Froio S, Formenti P, De Giorgis V, Galanti V, et al. Effect of mechanical power on intensive care mortality in ARDS patients. Crit Care. 2020;24:246. 32448389 10.1186/s13054-020-02963-x PMC7245621

[26] Pozzi T, Fratti I, Tomarchio E, Bruno G, Catozzi G, Monte A, et al. Early time-course of respiratory mechanics, mechanical power and gas exchange in ARDS patients. J Crit Care. 2024;79:154444. 37862955 10.1016/j.jcrc.2023.154444

[27] EL Costa V, Slutsky AS, Brochard LJ, Brower R, Serpa-Neto A, Cavalcanti AB, et al. Ventilatory Variables and Mechanical Power in Patients with Acute Respiratory Distress Syndrome. Am J Respir Crit Care Med. 2021;204:303–11. 33784486 10.1164/rccm.202009-3467OC

[28] Tharp WG, Neilson MR, Breidenstein MW, et al. Effects of obesity, pneumoperitoneum, and body position on mechanical power of intraoperative ventilation: an observational study. J Appl Physiol. 2023;134:1390–402. 37022962 10.1152/japplphysiol.00551.2022 PMC10211461

[29] Rocco PRM, Silva PL, Samary CS, et al. Elastic power but not driving power is the key promoter of ventilator-induced lung injury in experimental acute respiratory distress syndrome. Crit Care. 2020;24:284. 32493362 10.1186/s13054-020-03011-4 PMC7271482

[30] Lee HJ, Kim KS, Jeong JS, et al. Optimal positive end-expiratory pressure during robot-assisted laparoscopic radical prostatectomy. Korean J Anesthesiol. 2013;65:244. 24101959 10.4097/kjae.2013.65.3.244 PMC3790036

[31] Blecha S, Hager A, Gross V, et al. Effects of Individualised High Positive End-Expiratory Pressure and Crystalloid Administration on Postoperative Pulmonary Function in Patients Undergoing Robotic-Assisted Radical Prostatectomy: A Prospective Randomised Single-Blinded Pilot Study. J Clin Med. 2023;12:1460. 36835995 10.3390/jcm12041460 PMC9960679

[32] Cheng M, Ni L, Huang L, et al. Effect of positive end-expiratory pressure on pulmonary compliance and pulmonary complications in patients undergoing robot-assisted laparoscopic radical prostatectomy: a randomized control trial. BMC Anesthesiol. 2022;22:347. 36371148 10.1186/s12871-022-01869-1 PMC9652925

[33] Yoon H-K, Kim BR, Yoon S, et al. The Effect of Ventilation with Individualized Positive End-Expiratory Pressure on Postoperative Atelectasis in Patients Undergoing Robot-Assisted Radical Prostatectomy: A Randomized Controlled Trial. J Clin Med. 2021;10:850. 33669526 10.3390/jcm10040850 PMC7922101

[34] Zhou J, Wang C, Lv R, et al. Protective mechanical ventilation with optimal PEEP during RARP improves oxygenation and pulmonary indexes. Trials. 2021;22:351. 34011404 10.1186/s13063-021-05310-9 PMC8135157

[35] Young CC, Harris EM, Vacchiano C, Bodnar S, Bukowy B, Elliott RRD, et al. Lung-protective ventilation for the surgical patient: international expert panel-based consensus recommendations. Br J Anaesth. 2019;123:898–913. 31587835 10.1016/j.bja.2019.08.017

[36] Gutt CN, Oniu T, Mehrabi A, Kashfi A, Schemmer P, Büchler MW. Robot-assisted abdominal surgery. Br J Surg. 2004;91:1390–7. 15386325 10.1002/bjs.4700

[37] Covotta M, Claroni C, Torregiani G, et al. A Prospective, Randomized, Clinical Trial on the Effects of a Valveless Trocar on Respiratory Mechanics During Robotic Radical Cystectomy: A Pilot Study. Anesth Analg. 2017;124:1794–801. 28452822 10.1213/ANE.0000000000002027

[38] Horstmann M, Horton K, Kurz M, et al. Prospective Comparison Between the AirSeal® System Valve-Less Trocar and a Standard Versaport™ Plus V2 Trocar in Robotic-Assisted Radical Prostatectomy. J Endourol. 2013;27:579–82. 23186377 10.1089/end.2012.0632

